# Supplementary material for: Genome-Wide Analysis of the DNA Methylation Profile Identifies the fragile histidine triad (FHIT) Gene as a New Promising Biomarker of Crohn’s Disease
Source: J Clin Med. 2020 May 4;9(5):1338. doi: 10.3390/jcm9051338 (PMC7291297; doi:10.3390/jcm9051338)

Supplemental Table S1. List of differentially methylated CpG sites

DMRs	cgid	chr	start	end	gene	nmid	annot	cpg	prom	pval	adj. pval	FC_avg	nc_avg	cd_avg
Hyper	cg12827637	chr14	69256790	69256791	ZFP36L1	NM_004926	Body	Island	.	5.76E-05	0.000989714	4.465355492	0.06419878	0.287016909
Hyper	cg02072495	chr15	60689284	60689285	ANXA2	NM_001136015	5'UTR	N_Shore	.	2.04E-05	0.000560282	3.808196653	0.056572464	0.215719887
Hyper	cg26147845	chr12	132433837	132433838	EP400	NM_015409	TSS150(	N_Shore	Promoter	3.92E-06	0.000301217	3.643351981	0.077868629	0.283967159
Hyper	cg23737061	chr3	60226327	60226328	FHIT	NM_001166243	Body		.	1.65E-07	0.000239667	3.616168212	0.093920309	0.339893251
Hyper	cg18215449	chr12	66089472	66089473	.	.	.		.	4.22E-07	0.000239667	3.565225234	0.064067475	0.228671502
Hyper	cg24082121	chr5	672871	672872	TPPP	NM_007030	Body		.	1.78E-05	0.000525064	3.505843071	0.089095718	0.312606189
Hyper	cg23032421	chr3	3152037	3152038	IL5RA	NM_175727	5'UTR		.	1.04E-05	0.00041338	3.371418731	0.114213604	0.385299026
Hyper	cg11737172	chr8	1923051	1923052	KBTBD11	NM_014867	5'UTR	S_Shore	.	1.38E-05	0.000466865	3.368483202	0.108495063	0.365700645
Hyper	cg16886175	chr7	114562062	114562063	MDFIC	NM_001166345	TSS200	N_Shore	Promoter	7.28E-07	0.000243374	3.35638256	0.101463003	0.340784291
Hyper	cg10986412	chr5	672909	672910	TPPP	NM_007030	Body		.	1.56E-06	0.000255546	3.316242102	0.071981379	0.238939304
Hyper	cg02025737	chr15	33384750	33384751	.	.	.		.	3.80E-05	0.000776402	3.312390381	0.13643852	0.452168879
Hyper	cg06243675	chr8	11801364	11801365	.	.	.		.	5.23E-05	0.000932639	3.254014485	0.016251643	0.053108482
Hyper	cg09577804	chr10	3235531	3235532	.	.	.		.	5.04E-05	0.000912242	3.247042327	0.043496295	0.141459016
Hyper	cg23057220	chr19	1356314	1356315	MUM1	NR_024247	TSS200	S_Shore	Promoter	3.65E-05	0.000758178	3.204370027	0.14549078	0.466426732
Hyper	cg06984883	chr1	1243563	1243564	PUSL1	NM_153339	TSS150(	Island	Promoter	5.07E-06	0.000323878	3.198831858	0.005047892	0.01636724
Hyper	cg15963463	chr1	25253236	25253237	RUNX3	NM_001031680	Body	N_Shelf	.	5.64E-05	0.000977514	3.18669621	0.078493204	0.250352667
Hyper	cg06653052	chr19	1275166	1275167	C19orf24	NM_017914	TSS150(	Island	Promoter	3.34E-06	0.000290989	3.184250672	0.048706609	0.155312479
Hyper	cg09511741	chr19	49668728	49668729	TRPM4	NM_017636	Body	N_Shore	.	2.38E-05	0.000605015	3.165186894	0.191718252	0.607040616
Hyper	cg03707168	chr19	49379126	49379127	PPP1R15A	NM_014330	Body	S_Shelf	.	1.40E-05	0.000469682	3.157743559	0.071363261	0.225562653
Hyper	cg08255475	chr16	88871328	88871329	CDT1	NM_030928	Body	N_Shore	.	4.16E-06	0.000305647	3.100866245	0.103590812	0.32143134
Hyper	cg08591265	chr17	56082866	56082867	SFRS1	NM_001078166	3'UTR	N_Shore	.	3.33E-05	0.000719045	3.094495159	0.119656568	0.370486121
Hyper	cg10835083	chr2	222395100	222395101	EPHA4	NM_004438	Body		.	2.78E-05	0.000654934	3.037205824	0.128047198	0.389109418
Hyper	cg23422156	chr12	113587512	113587513	CCDC42B	NM_001144872	TSS200	N_Shelf	Promoter	1.80E-05	0.000527415	3.034134451	0.059533407	0.180835773
Hyper	cg12946660	chr19	41771872	41771873	HNRNPUL1	NM_007040	Body	S_Shore	.	5.57E-06	0.00033242	3.004275681	0.069969937	0.210409408
hypo	cg26994377	chr4	140579068	140579069	.	.	.		.	2.29E-05	0.000594729	0.33304523	0.437705704	0.145709102
hypo	cg07068735	chr15	63504870	63504871	RAB8B	NM_016530	Body		.	1.52E-06	0.000254329	0.332331187	0.533329303	0.177175193
hypo	cg14020652	chr9	38339334	38339335	.	.	.		.	6.32E-06	0.00034625	0.331950899	0.329875467	0.109435653
hypo	cg15488794	chr20	30262898	30262899	BCL2L1	NM_138578	Body		.	1.38E-05	0.000467058	0.331376467	0.404630606	0.134018198
hypo	cg01106881	chr2	220227421	220227422	.	.	.	S_Shelf	.	5.36E-05	0.000945982	0.328808209	0.390185823	0.128229182
hypo	cg02298094	chr12	111844675	111844676	SH2B3	NM_005475	5'UTR	S_Shore	.	2.10E-05	0.000568226	0.328760667	0.24262586	0.079698716
hypo	cg02017450	chr10	10631669	10631670	.	.	.		.	2.64E-05	0.000638148	0.328034436	0.516639432	0.169408328
hypo	cg14554869	chr5	74231170	74231171	.	.	.		.	4.55E-05	0.000858679	0.328017373	0.383287708	0.125657829
hypo	cg12433486	chr15	72520803	72520804	PKM2	NM_182470	5'UTR	N_Shore	.	1.30E-06	0.000248521	0.327812019	0.420371064	0.137735468
hypo	cg16465027	chr6	12595018	12595019	.	.	.		.	7.67E-06	0.000367097	0.327347357	0.372313534	0.121808586
hypo	cg05377162	chr1	185286995	185286996	IVNS1ABP	NM_006469	TSS150(	S_Shore	Promoter	3.73E-05	0.000767037	0.326673169	0.215948271	0.070477173
hypo	cg17879722	chr21	43181728	43181729	RIPK4	NM_020639	Body		.	5.14E-05	0.000922214	0.326629869	0.451177803	0.14730081
hypo	cg27543214	chr6	30761827	30761828	.	.	.		.	4.38E-06	0.000309823	0.324647143	0.615063463	0.19961106
hypo	cg16487653	chr16	73200704	73200705	.	.	.		.	1.56E-05	0.000493669	0.324411982	0.587421117	0.19049889
hypo	cg20907136	chr6	33041220	33041221	HLA-DPA1	NM_033554	Body	.	.	8.85E-06	0.000388122	0.323438432	0.358864026	0.116002762
hypo	cg10550882	chr20	1825646	1825647	.	.	.		.	3.85E-05	0.000781459	0.322783948	0.650603134	0.209936527
hypo	cg09657615	chr6	128921651	128921652	.	.	.		.	3.49E-06	0.000294266	0.322618251	0.399382461	0.128780333
hypo	cg02983090	chr16	27437891	27437892	IL21R	NM_021798	TSS1500		Promoter	1.89E-06	0.000260866	0.322522648	0.322494527	0.103944041
hypo	cg09657963	chr11	124790874	124790875	HEPACAM	NM_152722	3'UTR	Island	.	5.79E-05	0.000993027	0.322494146	0.243816674	0.078561699
hypo	cg20836412	chr8	82543284	82543285	.	.	.	Island	.	1.39E-05	0.000468249	0.321873353	0.278270281	0.089499976
hypo	cg01329005	chr19	17516711	17516712	BST2	NM_004335	TSS1500		Promoter	2.75E-05	0.00065198	0.321671343	0.535906455	0.172317916
hypo	cg20040488	chr6	28956509	28956510	.	.	.	Island	.	4.66E-05	0.000870721	0.321134706	0.269186903	0.08637737

hypo	cg22723502	chr6	26240527	26240528	HIST1H4F	NM_003540	TSS200	N_Shore	Promoter	2.79E-05	0.000656034	0.319269998	0.417719238	0.133297147
hypo	cg03367387	chr3	197439333	197439334	KIAA0226	NM_014687	Body		.	1.20E-05	0.000439301	0.31678741	0.585824878	0.185513625
hypo	cg05675514	chr6	22026014	22026015	FLJ22536	NR_015410	Body		.	2.71E-05	0.000647666	0.316737937	0.496961917	0.157338366
hypo	cg00134210	chr10	14644131	14644132	FAM107B	NM_031453	Body	N_Shore	.	4.75E-05	0.000880757	0.316201313	0.494021101	0.156141741
hypo	cg07636095	chr6	27759816	27759817	.	.	.		.	3.04E-06	0.000285718	0.313937292	0.279937941	0.087814353
hypo	cg03946324	chr10	14995580	14995581	DCLRE1C	NM_001033855	Body	N_Shore	.	6.52E-06	0.000348949	0.313694734	0.169935835	0.053239346
hypo	cg22413056	chr6	30650201	30650202	KIAA1949	NM_001134870	Body		.	5.77E-05	0.000990921	0.312736374	0.394933586	0.123441371
hypo	cg16250754	chr5	40841578	40841579	CARD6	NM_032587	1stExon		.	1.14E-06	0.000247212	0.311710708	0.463314598	0.144351292
hypo	cg24033661	chr11	2920054	2920055	SLC22A18AS	NM_007105	Body	N_Shelf	Promoter	6.89E-06	0.000354887	0.311358381	0.411747771	0.128132255
hypo	cg27093944	chr2	242908217	242908218	.	.	.		.	5.05E-05	0.00091326	0.311137169	0.528413131	0.164340079
hypo	cg10308253	chr6	149803110	149803111	ZC3H12D	NM_207360	5'UTR		.	5.67E-06	0.000334011	0.310988644	0.588854748	0.183058239
hypo	cg05336982	chr16	69564734	69564735	.	.	.		.	1.24E-06	0.000248311	0.310138254	0.286552302	0.088801844
hypo	cg18670236	chr3	126630477	126630478	CHCHD6	NM_032343	Body		.	3.57E-05	0.000747661	0.308820988	0.518550259	0.160070085
hypo	cg12081743	chr7	18535498	18535499	HDAC9	NM_058176	TSS1500		Promoter	1.82E-06	0.00025959	0.307457666	0.394115836	0.121104681
hypo	cg05956608	chr1	207999257	207999258	.	.	.		.	1.11E-06	0.000247212	0.305695743	0.289431259	0.088408473
hypo	cg06197966	chr10	29948323	29948324	SVIL	NM_003174	5'UTR		.	1.41E-07	0.000239667	0.305135075	0.386149756	0.117758348
hypo	cg12026563	chr19	18415803	18415804	.	.	.		.	2.88E-05	0.0006668	0.305079429	0.396196003	0.120801758
hypo	cg17822706	chr19	18415537	18415538	.	.	.		.	4.25E-07	0.000239667	0.304941407	0.280598332	0.085496545
hypo	cg19526685	chr8	126963506	126963507	.	.	.		Promoter	2.01E-06	0.000263911	0.304881783	0.541212555	0.164936337
hypo	cg08946844	chr12	110511111	110511112	.	.	.		.	1.28E-06	0.000248311	0.301927474	0.233426334	0.070408016
hypo	cg15716680	chr11	12864036	12864037	TEAD1	NM_021961	Body		.	3.49E-06	0.000294206	0.300878979	0.367956741	0.110640537
hypo	cg12956688	chr19	18415645	18415646	.	.	.		.	1.62E-06	0.000255546	0.300788002	0.361300725	0.108605002
hypo	cg24547575	chr6	31831680	31831681	NEU1	NM_000434	TSS150(	S_Shore	Promoter	3.24E-05	0.000708487	0.29814365	0.310970972	0.092643835
hypo	cg21193484	chr14	61938891	61938892	PRKCH	NM_006255	Body		.	3.16E-05	0.000699076	0.297531417	0.554767036	0.164990376
hypo	cg11706729	chr6	32813153	32813154	PSMB8	NM_004159	TSS150(	S_Shore	Promoter	2.60E-07	0.000239667	0.296745406	0.26786038	0.079416012
hypo	cg21571658	chr15	70785398	70785399	.	.	.		.	1.71E-05	0.000515611	0.29615317	0.306327908	0.090649596
hypo	cg04786142	chr1	234908380	234908381	.	.	.		.	1.78E-05	0.000524329	0.295515316	0.608121119	0.179638656
hypo	cg07474842	chr6	136915087	136915088	MAP3K5	NM_005923	Body		.	9.55E-07	0.000245873	0.292378394	0.509299601	0.148837437
hypo	cg25296938	chr7	98976899	98976900	ARPC1B	NM_005720	5'UTR	S_Shelf	.	6.25E-06	0.000344798	0.292284788	0.429242431	0.125390262
hypo	cg06202585	chr2	38325801	38325802	.	.	.		.	1.52E-05	0.000487715	0.291473542	0.428756192	0.124900233
hypo	cg13367647	chr1	155213713	155213714	GBA	NM_001005742	5'UTR		.	4.71E-05	0.000875671	0.291202152	0.357684696	0.104087673
hypo	cg03796321	chr15	86162441	86162442	AKAP13	NM_007200	Body		Promoter	5.73E-06	0.000335583	0.290904159	0.494266309	0.143713215
hypo	cg20019410	chr2	207939049	207939050	.	.	.		.	3.55E-05	0.000744957	0.289784231	0.403745391	0.116928026
hypo	cg15575880	chr6	33041217	33041218	HLA-DPA1	NM_033554	Body	.	.	8.17E-06	0.000376817	0.286273868	0.466488156	0.133471996
hypo	cg24366211	chr11	68610705	68610706	CPT1A	NM_001031847	TSS150(	N_Shore	Promoter	5.21E-06	0.000326665	0.285018131	0.303144663	0.086330227
hypo	cg09284066	chr7	25892406	25892407	.	.	.	Island	.	5.61E-06	0.00033288	0.28462655	0.242324143	0.068900347
hypo	cg05109049	chr17	29641332	29641333	NF1	NM_001042492	Body		Promoter	1.68E-05	0.000511441	0.283612522	0.394599792	0.111841803
hypo	cg01889574	chr11	44590888	44590889	CD82	NM_002231	5'UTR	S_Shelf	.	1.09E-05	0.000420726	0.283195394	0.329063303	0.093117531
hypo	cg17528580	chr10	69962885	69962886	MYPN	NM_032578	Body		.	1.48E-06	0.000254329	0.280320625	0.41083744	0.11509424
hypo	cg07643912	chr19	50819173	50819174	KCNC3	NM_004977	3'UTR		.	5.77E-05	0.000991601	0.279946911	0.394163744	0.110272917
hypo	cg27627038	chr6	475037	475038	.	.	.		.	1.68E-05	0.000510809	0.278722601	0.273943302	0.076282062
hypo	cg02293936	chr17	46671297	46671298	LOC404266	NR_033203	Body	Island	Promoter	1.14E-05	0.00042859	0.278577569	0.508672875	0.14163271
hypo	cg20294319	chr13	47253841	47253842	LRCH1	NM_015116	Body		.	6.85E-06	0.000354451	0.277992368	0.551359538	0.153201543
hypo	cg13379827	chr1	65468781	65468782	.	.	.	Island	.	2.37E-06	0.000271971	0.277188769	0.253422455	0.070173577
hypo	cg23721586	chr1	92197530	92197531	TGFBR3	NM_003243	Body		.	3.33E-06	0.000290742	0.276316205	0.395403592	0.109184052
hypo	cg12578486	chr13	100008956	100008957	UBAC2	NM_001144072	Body		.	1.40E-05	0.000469247	0.274448109	0.568779455	0.156027891
hypo	cg09701880	chr2	112252253	112252254	LOC541471	NR_015395	Body	.	.	1.87E-05	0.000537049	0.271417341	0.571770914	0.155115683
hypo	cg20175702	chr15	70053397	70053398	.	.	.		.	2.40E-05	0.000608128	0.271044343	0.456011232	0.123526369
hypo	cg11228682	chr6	16320529	16320530	ATXN1	NM_001128164	Body		.	2.48E-05	0.00061879	0.271013004	0.574352884	0.155584202
hypo	cg19738463	chr6	31831573	31831574	NEU1	NM_000434	TSS150(	S_Shore	Promoter	1.04E-06	0.000247212	0.268996178	0.415410168	0.111670647
hypo	cg17837069	chr10	30138587	30138588	.	.	.		.	3.03E-07	0.000239667	0.268595668	0.29562553	0.079330596
hypo	cg13995427	chr9	117134860	117134861	AKNA	NM_030767	Body		.	6.09E-06	0.000341589	0.26283093	0.603633479	0.158579831

hypo	cg20893203	chr6	41499798	41499799	.	.	.		.	2.58E-05	0.000629618	0.261221932	0.558334688	0.145775388
hypo	cg06747543	chr19	18589893	18589894	ELL	NM_006532	Body		.	1.96E-06	0.000263494	0.258885444	0.312125928	0.080730748
hypo	cg11214507	chr1	210419937	210419938	.	.	.		.	6.84E-07	0.000243374	0.256361424	0.507538081	0.130038822
hypo	cg07218288	chr6	32813210	32813211	PSMB8	NM_004159	TSS150(	S_Shore	Promoter	2.99E-08	0.000239667	0.253344702	0.28086197	0.071080227
hypo	cg06719604	chr2	55361309	55361310	.	.	.		.	1.39E-06	0.000249807	0.248561831	0.374783771	0.093081797
hypo	cg07201215	chr21	36419477	36419478	RUNX1	NM_001754	Body		.	1.43E-07	0.000239667	0.248488664	0.201808128	0.050071881
hypo	cg16754364	chr16	29677968	29677969	SPN	NM_001030288	3'UTR	S_Shore	.	1.60E-05	0.000499615	0.248465852	0.238576419	0.05920294
hypo	cg11558551	chr19	17516441	17516442	BST2	NM_004335	TSS200		Promoter	2.01E-09	0.000239667	0.246495963	0.245665322	0.06048016
hypo	cg05876246	chr8	81831078	81831079	.	.	.		.	6.16E-06	0.000342824	0.245951258	0.521927636	0.128293354
hypo	cg19308375	chr2	228324936	228324937	.	.	.		.	3.95E-07	0.000239667	0.24589247	0.577058807	0.141819005
hypo	cg18496653	chr21	38593041	38593042	.	.	.	Island	.	1.24E-05	0.000445335	0.243717811	0.198485515	0.048298827
hypo	cg23145794	chr20	20433061	20433062	RALGAPA2	NM_020343	Body	N_Shore	.	3.72E-06	0.00029824	0.242960579	0.377592213	0.091664319
hypo	cg22047295	chr2	175462521	175462522	WIPF1	NM_003387	5'UTR		.	2.66E-05	0.000640751	0.24208896	0.510790931	0.123581054
hypo	cg13786863	chr11	68610742	68610743	CPT1A	NM_001031847	TSS150(	N_Shore	Promoter	1.80E-05	0.000526916	0.236940384	0.349882827	0.082825065
hypo	cg10924691	chr14	21439547	21439548	.	.	.	N_Shore	.	2.81E-05	0.000658196	0.235379208	0.370760548	0.087192862
hypo	cg24626312	chr17	46671331	46671332	LOC404266	NR_033203	Body	Island	Promoter	1.47E-05	0.000480538	0.234897211	0.31718163	0.07442857
hypo	cg10153335	chr17	46671292	46671293	LOC404266	NR_033203	Body	Island	Promoter	4.61E-05	0.000865221	0.23257743	0.548864068	0.127576652
hypo	cg06061086	chr6	41499639	41499640	.	.	.		.	4.80E-07	0.000239667	0.232067185	0.589502752	0.136727451
hypo	cg14043253	chr8	81830960	81830961	.	.	.		.	9.83E-07	0.000245873	0.230830483	0.459454722	0.105979238
hypo	cg07706715	chr1	161510193	161510194	.	.	.		.	3.82E-05	0.000778723	0.227783781	0.246691791	0.056115167
hypo	cg15583014	chr1	65468658	65468659	.	.	.	Island	.	1.52E-06	0.000254329	0.227269846	0.130263614	0.029527719
hypo	cg03071808	chr2	242908130	242908131	.	.	.		.	1.49E-05	0.000484257	0.226565848	0.39433008	0.089264386
hypo	cg03130910	chr1	234908225	234908226	.	.	.		.	1.65E-05	0.000507903	0.226520376	0.482815951	0.109290303
hypo	cg22806907	chr17	40826211	40826212	PLEKHH3	NM_024927	Body	N_Shore	.	2.24E-05	0.000587229	0.225625672	0.402610905	0.090761919
hypo	cg24203709	chr15	42187178	42187179	SPTBN5	NM_016642	TSS1500		Promoter	4.68E-09	0.000239667	0.224255906	0.290623865	0.065096544
hypo	cg10087036	chr16	19130460	19130461	ITPRIPL2	NM_001034841	1stExon	S_Shelf	.	4.48E-05	0.000852534	0.220522499	0.467774097	0.103076765
hypo	cg20245757	chr17	46663758	46663759	.	.	.	S_Shelf	.	9.65E-06	0.000401459	0.220353615	0.395667189	0.087108731
hypo	cg26590411	chr4	119199645	119199646	SNORA24	NR_002963	TSS150(	Island	Promoter	2.56E-06	0.000274886	0.214515333	0.10825681	0.023144197
hypo	cg02413040	chr21	36419647	36419648	RUNX1	NM_001754	Body		.	2.74E-07	0.000239667	0.214176618	0.303609169	0.064947403
hypo	cg24345715	chr19	13898672	13898673	.	.	.		.	7.87E-07	0.000244182	0.205171778	0.287400073	0.058886901
hypo	cg25320388	chr11	6767765	6767766	.	.	.		.	2.53E-05	0.000623235	0.202850913	0.443729615	0.089931243
hypo	cg11623922	chr6	27759385	27759386	.	.	.		.	7.34E-06	0.000362122	0.201784135	0.378677731	0.076331337
hypo	cg22491409	chr12	125198294	125198295	.	.	.		.	2.68E-06	0.000277493	0.180898922	0.476188427	0.086060063
hypo	cg14541915	chr11	65894462	65894463	PACS1	NM_018026	Body		.	2.74E-06	0.000278759	0.175066438	0.400649064	0.070057711
hypo	cg13475583	chr1	235116730	235116731	.	.	.		.	7.29E-06	0.000361207	0.173215444	0.333758596	0.057729465
hypo	cg00455424	chr1	65468814	65468815	.	.	.	Island	.	2.82E-05	0.0006595	0.17063815	0.20394024	0.034717049
hypo	cg24166450	chr17	74248465	74248466	.	.	.		.	6.82E-08	0.000239667	0.165090835	0.271397918	0.044721818

Supplemental Table S2. Primer information in this study

Gene	Target	Methylation specific PCR	
		Forward Primer (5'-3')	Reverse Primer (5'-3')
TRPM4	Unmethylation	TTTTGGGTTGTAGGAGGTTGT	ACCCAAAAATCTAAATATCCAACAC
	Methylation	TTTGGGTTGTAGGAGGTTGC	ACCCAAAAATCTAAATATCCAACG
MUM1	Unmethylation	ATTTTGTTTTAGGATATATTGGTGT	CAAAATAATAACTATCACCTTCACA
	Methylation	AAATTTCGTTTTAGGATATATTGGC	CAAAATAATAACTATCACCTTCGCA
EPHA4	Unmethylation	TATTTATTTTATTGGGATAGAAGTGG	CCCTAAAACCTATAACAACATCACA
	Methylation	TATTTATTTTATTGGGATAGAAGCGG	TACCCTAAAACCTATAACAACGTCG
SFRS1	Unmethylation	GTTGTAGAGTATGGTGGGATAATGT	AATCAAATCTTAAAAAATAATCAAT
	Methylation	GTCGTAGAGTATGGTGGGATAAC	AATCGAATCTTAAAAAATAATCGAT
MDFIC	Unmethylation	GGGTTAAGGTTTTAAATGTATATGT	AAAACTTTAAAAATATCCTAACAAA
	Methylation	AGGGGTTAAGGTTTTAAACGTATAC	AAAACTTTAAAAATATCCTAACGAA
FHIT	Unmethylation	AGAAATTTAGTTAGTGGGAAGTTGT	AAAAAAAATTTAAAACATAAATCACA
	Methylation	AGAAATTTAGTTAGTGGGAAGTCGT	AAAAAAAATTTAAAACATAAATCGCA
CDT1	Unmethylation	TTAAGAGAGTGGATTATTGAATTGG	ACCTAAAAATAACATAAACCCCATA
	Methylation	TTTTAAGAGAGAGCGGATTATTGAATC	CCTAAAAATAACGTAAACCCCGTA
TPPP	Unmethylation	GTGGTTGTGTTTATTTATTTTTTGT	AAAACCTCTACCTTACAAAACCAAC
	Methylation	CGGTCGCGTTTATTTATTTTTC	GAAACCTCTACCTTACAAAACCGA
ZFP36L1	Unmethylation	TTTTAGTTTGGAGTTAGAAAGGTGA	AATCAAAAATAATAACAAACACAAA
	Methylation	GTTTTAGTTCGGAGTTAGAAAGGC	AATCGAAAATAATAACAAACACGAA
EP400	Unmethylation	ATTTTAAATGATTTTTTTGTTTTGG	AATTACATATATAAACCACCACACC
	Methylation	TGATTTTAAATGATTTTTTCGTTTC	AAATTACATATATAAACCACCACGC
RUNX3	Unmethylation	TTACGAGGGGCGGTCGTACGCGGG	AAAACGACCGACGCGAACGCCTCC
	Methylation	TTATGAGGGGTGGTTGTATGTGGG	AAAACAACCAACACAAACACCTCC
KBTBD11	Unmethylation	TTTGGTATTTATGGAAATTTTATTGT	ATATAACCTCTACCTCCCACTCAAA
	Methylation	TTTCGGTATTTATGGAAATTTTATC	AATATAACCTCTACCTCCCACTCG
PPP1R15A	Unmethylation	TTTGATTAATATGGAGAAATTATGT	CTCAAACTAAAATACAAAAAAACAAT
	Methylation	TTTGATTAATATGGAGAAATTACGT	TCAAACTAAAATACAAAAAAACGAT
ANXA2	Unmethylation	TATTGAGTAAGGGAGTTGGTTAAATG	ATAACTTTAAACCCAAAATTTCATA
	Methylation	TATCGAGTAAGGGAGTTGGTTAAAC	ATAACTTTAAACCCGAAATTTCGTA
HNRNPUL1	Unmethylation	TAGTTTGATTTATGGGAATAGTTGA	AAAAAACAAATTATAAAAAACACAAC
	Methylation	GATAGTTCGATTTACGGGAATAGTC	ACGAAAAACAAATTATAAAAAACACG
C19orf24	Unmethylation	GGGGTGGTTAGTTTTTGTTTATAAT	TCTTCCCTATCAAATCCTACTCATA
	Methylation	GGGCGGTTAGTTTTTGTTTATAAC	TTCCCTATCGAATCCTACTCGTA
PUSL1	Unmethylation	ATTTTGTTTTAGGATATATTGGTGT	CAAAATAATAACTATCACCTTCACA
	Methylation	AAATTTCGTTTTAGGATATATTGGC	CAAAATAATAACTATCACCTTCGCA
Alu	Methylation	ATTAGTCGGGCGTGGTGG	CCCGAATTCAAACGATTCTCC

Gene	locus	Bisulfite sequencing	
		Forward Primer (5'-3')	Reverse Primer (5'-3')
FHIT	Chr3 (59,747,277-61,251,452)	TTAGAAAGATTTAGAGTGGGGAAAT	CCCCTTACAAAAAAATTTAAA ACATAA

Supplemental Table S3. List of hypermethylated genes for gene network analysis (related to Figure 5)

Input ID	Gene ID	Gene Symbol	Description	Biological Process (GO)
ZFP36L1	677	ZFP36L1	ZFP36 ring finger protein like 1	GO:0045657 positive regulation of monocyte differentiation;<br>GO:0097403 cellular response to raffinose;GO:0060710 chorio-allantoic fusion
ANXA2	302	ANXA2	annexin A2	GO:0052362 catabolism by host of symbiont protein;GO:1905602 positive regulation<br>of receptor-mediated endocytosis involved in cholesterol transport;GO:0052417 metabolism by host of symbiont protein
EP400	57634	EP400	E1A binding protein p400	GO:0043968 histone H2A acetylation;GO:0043967 histone H4 acetylation;GO:0016573 histone acetylation
FHIT	2272	FHIT	fragile histidine triad diadenosine triphosphatase	GO:0032435 negative regulation of proteasomal ubiquitin-dependent protein catabolic process;<br>GO:2000059 negative regulation of ubiquitin-dependent protein catabolic process;GO:1901799 negative regulation of proteasomal protein catabolic process
TPPP	11076	TPPP	tubulin polymerization promoting protein	GO:0046785 microtubule polymerization;GO:0032273 positive regulation of protein polymerization;GO:0001578 microtubule bundle formation
IL5RA	3568	IL5RA	interleukin 5 receptor subunit alpha	GO:0038043 interleukin-5-mediated signaling pathway;GO:0032674 regulation of interleukin-5 production;GO:0032634 interleukin-5 production
KBTBD11	9920	KBTBD11	kelch repeat and BTB domain containing 11	
MDFIC	29969	MDFIC	MyoD family inhibitor domain containing	GO:0007257 activation of JUN kinase activity;GO:0042308 negative regulation of protein import into nucleus;GO:0050434 positive regulation of viral transcription
MUM1	3662	IRF4	interferon regulatory factor 4	GO:0072540 T-helper 17 cell lineage commitment;GO:0002295 T-helper cell lineage commitment;GO:0072539 T-helper 17 cell differentiation
PUSL1	126789	PUSL1	pseudouridine synthase like 1	GO:0031119 tRNA pseudouridine synthesis;GO:0001522 pseudouridine synthesis;GO:0006400 tRNA modification
RUNX3	864	RUNX3	RUNX family transcription factor 3	GO:0043378 positive regulation of CD8-positive, alpha-beta T cell differentiation;<br>GO:0043376 regulation of CD8-positive, alpha-beta T cell differentiation;GO:0043371 negative regulation of CD4-positive, alpha-beta T cell differentiation
C19orf24	55009	FAM174C	family with sequence similarity 174 member C	
TRPM4	54795	TRPM4	transient receptor potential cation<br>channel subfamily M member 4	GO:1903949 positive regulation of atrial cardiac muscle cell action potential;<br>GO:0086047 membrane depolarization during Purkinje myocyte cell action potential;GO:0086048 membrane depolarization during bundle of His cell action potential
PPP1R15A	23645	PPP1R15A	protein phosphatase 1 regulatory subunit 15A	GO:1903917 positive regulation of endoplasmic reticulum stress-induced eIF2 alpha dephosphorylation;GO:1903916 regulation of<br>endoplasmic reticulum stress-induced eIF2 alpha dephosphorylation;GO:0036497 eIF2alpha dephosphorylation in response to endoplasmic reticulum stress
CDT1	81620	CDT1	chromatin licensing and DNA replication factor 1	GO:1902426 deactivation of mitotic spindle assembly checkpoint;GO:0090266 regulation of mitotic cell cycle spindle assembly checkpoint;<br>GO:1903504 regulation of mitotic spindle checkpoint
SFRS1	6426	SRSF1	serine and arginine rich splicing factor 1	GO:0000395 mRNA 5'-splice site recognition;GO:0006406 mRNA export from nucleus;GO:0006376 mRNA splice site selection
EPHA4	2043	EPHA4	EPH receptor A4	GO:2001108 positive regulation of Rho guanyl-nucleotide exchange factor activity;GO:0021957 corticospinal tract morphogenesis;<br>GO:0097156 fasciculation of motor neuron axon
CCDC42B	387885	CFAP73	cilia and flagella associated protein 73	GO:0036159 inner dynein arm assembly;GO:0070286 axonemal dynein complex assembly;GO:2000574 regulation of microtubule motor activity
HNRNPUL1	11100	HNRNPUL1	heterogeneous nuclear ribonucleoprotein U like 1	GO:0000398 mRNA splicing, via spliceosome;GO:0009615 response to virus;GO:0000377 RNA splicing, via transesterification reactions with bulged adenosine as nucleophile
TRIM10	10107	TRIM10	tripartite motif containing 10	GO:0046597 negative regulation of viral entry into host cell;GO:0046596 regulation of viral entry into host cell;GO:0046718 viral entry into host cell
CACNA1C	775	CACNA1C	calcium voltage-gated channel subunit alpha1 C	GO:0010881 regulation of cardiac muscle contraction by regulation of the release of sequestered calcium ion;GO:0010880 regulation of release of<br>sequestered calcium ion into cytosol by sarcoplasmic reticulum;GO:0098912 membrane depolarization during atrial cardiac muscle cell action potential
RHPN2	85415	RHPN2	rhophilin Rho GTPase binding protein 2	GO:0007165 signal transduction;GO:0023052 signaling;GO:0007154 cell communication
SLMAP	7871	SLMAP	sarcolemma associated protein	GO:1905150 regulation of voltage-gated sodium channel activity;GO:1900825 regulation of membrane depolarization<br>during cardiac muscle cell action potential;GO:0098902 regulation of membrane depolarization during action potential
CYP46A1	10858	CYP46A1	cytochrome P450 family 46 subfamily A member 1	GO:0006707 cholesterol catabolic process;GO:1900271 regulation of long-term synaptic potentiation;GO:0042448 progesterone metabolic process
VEZF1	7716	VEZF1	vascular endothelial zinc finger 1	GO:0045603 positive regulation of endothelial cell differentiation;GO:0045601 regulation of endothelial cell differentiation;GO:0001885 endothelial cell development
FAM55B	120406	NXPE2	neurexophilin and PC-esterase<br>domain family member 2	
C9orf3	84909	AOPEP	aminopeptidase O (putative)	GO:0002003 angiotensin maturation;GO:0002002 regulation of angiotensin levels in blood;GO:0001991 regulation of systemic arterial blood pressure<br>by circulatory renin-angiotensin
LMF1	64788	LMF1	lipase maturation factor 1	GO:0051006 positive regulation of lipoprotein lipase activity;GO:0034382 chylomicron remnant clearance;GO:0061365 positive regulation of triglyceride lipase activity
MLXIP	22877	MLXIP	MLX interacting protein	GO:1900402 regulation of carbohydrate metabolic process by regulation of transcription from RNA polymerase II promoter;<br>GO:0006109 regulation of carbohydrate metabolic process;GO:0045944 positive regulation of transcription by RNA polymerase II
VAT1L	57687	VAT1L	vesicle amine transport 1 like	GO:0055114 oxidation-reduction process;GO:0008152 metabolic process;GO:0008150 biological_process
PDZRN3	23024	PDZRN3	PDZ domain containing ring finger 3	GO:0007528 neuromuscular junction development;GO:0016567 protein ubiquitination;GO:0050808 synapse organization

FAT1	2195	FAT1	FAT atypical cadherin 1	GO:0045197 establishment or maintenance of epithelial cell apical/basal polarity;GO:0003382 epithelial cell morphogenesis;GO:0048593 camera-type eye morphogenesis
SDK1	221935	SDK1	sidekick cell adhesion molecule 1	GO:0010842 retina layer formation;GO:0060998 regulation of dendritic spine development;GO:0048148 behavioral response to cocaine
KCNK10	54207	KCNK10	potassium two pore domain channel subfamily K member 10	GO:0030322 stabilization of membrane potential;GO:0071805 potassium ion transmembrane transport;GO:0007613 memory
MYO10	4651	MYO10	myosin X	GO:0038096 Fc-gamma receptor signaling pathway involved in phagocytosis; GO:0002431 Fc receptor mediated stimulatory signaling pathway;GO:0038094 Fc-gamma receptor signaling pathway
ATG12	9140	ATG12	autophagy related 12	GO:0006501 C-terminal protein lipidation;GO:0018410 C-terminal protein amino acid modification;GO:0000422 autophagy of mitochondrion
NUP210L	91181	NUP210L	nucleoporin 210 like	GO:0060009 Sertoli cell development;GO:0060008 Sertoli cell differentiation;GO:0007286 spermatid development
SPATA2L	124044	SPATA2L	spermatogenesis associated 2 like	
HOXA3	3200	HOXA3	homeobox A3	GO:0021615 glossopharyngeal nerve morphogenesis;GO:0021563 glossopharyngeal nerve development;GO:1900122 positive regulation of receptor binding
LOC201651	201651	AADACP1	arylacetamide deacetylase pseudogene 1	
KLK11	11012	KLK11	kallikrein related peptidase 11	GO:0006508 proteolysis;GO:0019538 protein metabolic process;GO:1901564 organonitrogen compound metabolic process
ABAT	18	ABAT	4-aminobutyrate aminotransferase	GO:1904450 positive regulation of aspartate secretion;GO:1904448 regulation of aspartate secretion;GO:0014053 negative regulation of gamma-aminobutyric acid secretion
CD63	967	CD63	CD63 molecule	GO:0035646 endosome to melanosome transport;GO:0043485 endosome to pigment granule transport;GO:0048757 pigment granule maturation
CDH22	64405	CDH22	cadherin 22	GO:0016339 calcium-dependent cell-cell adhesion via plasma membrane cell adhesion molecules; GO:0044331 cell-cell adhesion mediated by cadherin;GO:0007043 cell-cell junction assembly
FRMPD2	143162	FRMPD2	FERM and PDZ domain containing 2	GO:0070830 bicellular tight junction assembly;GO:0120192 tight junction assembly;GO:0043297 apical junction assembly
ADSSL1	122622	ADSS1	adenylosuccinate synthase 1	GO:0044208 'de novo' AMP biosynthetic process;GO:0006167 AMP biosynthetic process;GO:0006531 aspartate metabolic process
LIMA1	51474	LIMA1	LIM domain and actin binding 1	GO:0030299 intestinal cholesterol absorption;GO:0030835 negative regulation of actin filament depolymerization;GO:0098856 intestinal lipid absorption
PCGF2	7703	PCGF2	polycomb group ring finger 2	GO:0036353 histone H2A-K119 monoubiquitination;GO:0035518 histone H2A monoubiquitination;GO:0070317 negative regulation of G0 to G1 transition
TMEM87A	25963	TMEM87A	transmembrane protein 87A	GO:0042147 retrograde transport, endosome to Golgi;GO:0016482 cytosolic transport;GO:0016197 endosomal transport
PODXL2	50512	PODXL2	podocalyxin like 2	GO:0050901 leukocyte tethering or rolling;GO:0061756 leukocyte adhesion to vascular endothelial cell;GO:0045123 cellular extravasation
SLC9A2	6549	SLC9A2	solute carrier family 9 member A2	GO:0098719 sodium ion import across plasma membrane;GO:0051453 regulation of intracellular pH;GO:0030641 regulation of cellular pH
LIMCH1	22998	LIMCH1	LIM and calponin homology domains 1	GO:0051496 positive regulation of stress fiber assembly;GO:0051492 regulation of stress fiber assembly;GO:0051893 regulation of focal adhesion assembly
HOXA4	3201	HOXA4	homeobox A4	GO:0048704 embryonic skeletal system morphogenesis;GO:0048706 embryonic skeletal system development;GO:0048705 skeletal system morphogenesis
C9orf71	169693	TMEM252	transmembrane protein 252	
OSCP1	127700	OSCP1	organic solute carrier partner 1	GO:1990961 drug transmembrane export;GO:0046618 drug export;GO:0006855 drug transmembrane transport
TLX1NB	10003824	TLX1NB	TLX1 neighbor	
MACF1	23499	MACF1	microtubule actin crosslinking factor 1	GO:0045773 positive regulation of axon extension;GO:0051893 regulation of focal adhesion assembly;GO:0050772 positive regulation of axonogenesis
ADO	84890	ADO	2-aminoethanethiol dioxygenase	GO:0000098 sulfur amino acid catabolic process;GO:0000096 sulfur amino acid metabolic process;GO:0044273 sulfur compound catabolic process
UTS2D	257313	UTS2B	urotensin 2B	GO:0097746 regulation of blood vessel diameter;GO:0050880 regulation of blood vessel size;GO:0035296 regulation of tube diameter
CAPN13	92291	CAPN13	calpain 13	GO:0006508 proteolysis;GO:0019538 protein metabolic process;GO:1901564 organonitrogen compound metabolic process
C12orf72	254013	ETFBKMT	electron transfer flavoprotein subunit beta lysine methyltransferase	GO:1904736 negative regulation of fatty acid beta-oxidation using acyl-CoA dehydrogenase;GO:1904735 regulation of fatty acid beta-oxidation using acyl-CoA dehydrogenase;GO:0031999 negative regulation of fatty acid beta-oxidation
MTMR11	10903	MTMR11	myotubularin related protein 11	
MLST8	64223	MLST8	MTOR associated protein, LST8 homolog	GO:0032148 activation of protein kinase B activity;GO:1900034 regulation of cellular response to heat;GO:0032008 positive regulation of TOR signaling
WIZ	58525	WIZ	WIZ zinc finger	GO:0010571 positive regulation of nuclear cell cycle DNA replication;GO:0033262 regulation of nuclear cell cycle DNA replication; GO:2000105 positive regulation of DNA-dependent DNA replication
ARID5B	84159	ARID5B	AT-rich interaction domain 5B	GO:0060613 fat pad development;GO:0060325 face morphogenesis;GO:0008585 female gonad development
CISH	1154	CISH	cytokine inducible SH2 containing protein	GO:0046627 negative regulation of insulin receptor signaling pathway;GO:0038111 interleukin-7-mediated signaling pathway; GO:1900077 negative regulation of cellular response to insulin stimulus

MIR760	10012634[illegible]	MIR760	microRNA 760	GO:0035195 gene silencing by miRNA;GO:0035194 posttranscriptional gene silencing by RNA;GO:0016441 posttranscriptional gene silencing
AFAP1	60312	AFAP1	actin filament associated protein 1	GO:0051493 regulation of cytoskeleton organization;GO:0033043 regulation of organelle organization;GO:0007010 cytoskeleton organization
C6orf145	221749	PXDC1	PX domain containing 1	
PSORS1C1	170679	PSORS1C1	psoriasis susceptibility 1 candidate 1	
C17orf64	124773	C17orf64	chromosome 17 open reading frame 64	
MYLK	4638	MYLK	myosin light chain kinase	GO:0060414 aorta smooth muscle tissue morphogenesis;GO:0035909 aorta morphogenesis;GO:0071476 cellular hypotonic response
KIAA1026		None	None	None
EHD2	30846	EHD2	EH domain containing 2	GO:2001137 positive regulation of endocytic recycling;GO:1901741 positive regulation of myoblast fusion;GO:1901739 regulation of myoblast fusion
ALDH3A1	218	ALDH3A1	aldehyde dehydrogenase 3 family member A1	GO:0051591 response to cAMP;GO:0051384 response to glucocorticoid;GO:0006805 xenobiotic metabolic process
SLC22A11	55867	SLC22A11	solute carrier family 22 member 11	GO:0046415 urate metabolic process;GO:0015698 inorganic anion transport;GO:0015711 organic anion transport
TOM1L2	146691	TOM1L2	target of myb1 like 2 membrane trafficking protein	GO:0045839 negative regulation of mitotic nuclear division;GO:0051784 negative regulation of nuclear division;GO:0007088 regulation of mitotic nuclear division
ARHGAP12	94134	ARHGAP12	Rho GTPase activating protein 12	GO:0051058 negative regulation of small GTPase mediated signal transduction;GO:0002011 morphogenesis of an epithelial sheet;<br>GO:0006911 phagocytosis, engulfment
JMJD8	339123	JMJD8	jumonji domain containing 8	GO:1903302 regulation of pyruvate kinase activity;GO:0006110 regulation of glycolytic process;GO:1903672 positive regulation of sprouting angiogenesis
KTN1	3895	KTN1	kinectin 1	GO:0043687 post-translational protein modification;GO:0007018 microtubule-based movement;GO:0007017 microtubule-based process
LHCGR	3973	LHCGR	luteinizing hormone/choriogonadotropin receptor	GO:0032962 positive regulation of inositol trisphosphate biosynthetic process;GO:0032960 regulation of inositol trisphosphate biosynthetic process;<br>GO:0071373 cellular response to luteinizing hormone stimulus
RAD21	5885	RAD21	RAD21 cohesin complex component	GO:0045841 negative regulation of mitotic metaphase/anaphase transition;GO:0045876 positive regulation of sister chromatid cohesion;<br>GO:0030071 regulation of mitotic metaphase/anaphase transition
IFI27	3429	IFI27	interferon alpha inducible protein 27	GO:0044827 modulation by host of viral genome replication;GO:0044788 modulation by host of viral process;<br>GO:0046825 regulation of protein export from nucleus
KTELC1	56983	POGLUT1	protein O-glucosyltransferase 1	GO:0018242 protein O-linked glycosylation via serine;GO:0048318 axial mesoderm development;GO:0048339 paraxial mesoderm development
BCAS3	54828	BCAS3	BCAS3 microtubule associated cell migration factor	GO:0051895 negative regulation of focal adhesion assembly;GO:0051893 regulation of focal adhesion assembly;<br>GO:2000251 positive regulation of actin cytoskeleton reorganization
AGTR1	185	AGTR1	angiotensin II receptor type 1	GO:0002018 renin-angiotensin regulation of aldosterone production;GO:2000858 regulation of aldosterone secretion;GO:0035932 aldosterone secretion
FLJ41603		None	None	None
DEPDC5	9681	DEPDC5	DEP domain containing 5	GO:1904262 negative regulation of TORC1 signaling;GO:0034198 cellular response to amino acid starvation;GO:1903432 regulation of TORC1 signaling
ADD3	120	ADD3	adducin 3	GO:0051016 barbed-end actin filament capping;GO:0051693 actin filament capping;GO:0030835 negative regulation of actin filament depolymerization
POP7	10248	POP7	POP7 homolog, ribonuclease P/MRP subunit	GO:0001682 tRNA 5'-leader removal;GO:0099116 tRNA 5'-end processing;GO:0034471 ncRNA 5'-end processing
RAPGEF4	11069	RAPGEF4	Rap guanine nucleotide exchange factor 4	GO:0050796 regulation of insulin secretion;GO:0030073 insulin secretion;GO:0090276 regulation of peptide hormone secretion
IL22RA1	58985	IL22RA1	interleukin 22 receptor subunit alpha 1	GO:0050829 defense response to Gram-negative bacterium;GO:0042742 defense response to bacterium;GO:0019221 cytokine-mediated signaling pathway
PTK7	5754	PTK7	protein tyrosine kinase 7 (inactive)	GO:0090179 planar cell polarity pathway involved in neural tube closure;GO:0090178 regulation of establishment of planar polarity involved in neural tube closure<br>;GO:0090177 establishment of planar polarity involved in neural tube closure
UCN3	114131	UCN3	urocortin 3	GO:0032024 positive regulation of insulin secretion;GO:0051412 response to corticosterone;GO:0090277 positive regulation of peptide hormone secretion
TFCP2L1	29842	TFCP2L1	transcription factor CP2 like 1	GO:0002070 epithelial cell maturation;GO:0007431 salivary gland development;GO:0007028 cytoplasm organization
ANO4	121601	ANO4	anoctamin 4	GO:0061589 calcium activated phosphatidylserine scrambling;GO:0061590 calcium activated phosphatidylcholine scrambling<br>GO:0061591 calcium activated galactosylceramide scrambling
RBM47	54502	RBM47	RNA binding motif protein 47	GO:0016554 cytidine to uridine editing;GO:0016553 base conversion or substitution editing;GO:0002244 hematopoietic progenitor cell differentiation
WDR1	9948	WDR1	WD repeat domain 1	GO:0042247 establishment of planar polarity of follicular epithelium;GO:0030043 actin filament fragmentation;<br>GO:0030836 positive regulation of actin filament depolymerization
PPM1F	9647	PPM1F	protein phosphatase, Mg2+/Mn2+ dependent 1F	GO:0044387 negative regulation of protein kinase activity by regulation of protein phosphorylation;GO:0051894 positive regulation of focal adhesion assembly;<br>GO:0051496 positive regulation of stress fiber assembly
MPL	4352	MPL	MPL proto-oncogene, thrombopoietin receptor	GO:1905221 positive regulation of platelet formation;GO:1905219 regulation of platelet formation;GO:1990959 eosinophil homeostasis

SPRED2	200734	SPRED2	sprouty related EVH1 domain containing 2	GO:0000188 inactivation of MAPK activity;GO:0043517 positive regulation of DNA damage response, signal transduction by p53 class mediator;<br>GO:0010801 negative regulation of peptidyl-threonine phosphorylation
RNF13	11342	RNF13	ring finger protein 13	GO:0051865 protein autoubiquitination;GO:0006511 ubiquitin-dependent protein catabolic process;GO:0019941 modification-dependent protein catabolic process
LRRFIP1	9208	LRRFIP1	LRR binding FLII interacting protein 1	GO:0032481 positive regulation of type I interferon production;GO:0051092 positive regulation of NF-kappaB transcription factor activity;<br>GO:0032479 regulation of type I interferon production
C1R	715	C1R	complement C1r	GO:0006958 complement activation, classical pathway;GO:0030449 regulation of complement activation;<br>GO:0002455 humoral immune response mediated by circulating immunoglobulin
TEAD1	7003	TEAD1	TEA domain transcription factor 1	GO:1902895 positive regulation of pri-miRNA transcription by RNA polymerase II;GO:1902893 regulation of pri-miRNA transcription by RNA polymerase II;<br>GO:0061614 pri-miRNA transcription by RNA polymerase II
POLA2	23649	POLA2	DNA polymerase alpha 2, accessory subunit	GO:0032201 telomere maintenance via semi-conservative replication;GO:0033260 nuclear DNA replication;GO:0006270 DNA replication initiation
GPX5	2880	GPX5	glutathione peroxidase 5	GO:0098869 cellular oxidant detoxification;GO:1990748 cellular detoxification;GO:0098754 detoxification
CCDC57	284001	CCDC57	coiled-coil domain containing 57	
ARSA	410	ARSA	arylsulfatase A	GO:0043312 neutrophil degranulation;GO:0006687 glycosphingolipid metabolic process;GO:0002283 neutrophil activation involved in immune response
SIL1	64374	SIL1	SIL1 nucleotide exchange factor	GO:0006457 protein folding;GO:0006886 intracellular protein transport;GO:0015031 protein transport
EFHB	151651	EFHB	EF-hand domain family member B	GO:2001256 regulation of store-operated calcium entry;GO:0070884 regulation of calcineurin-NFAT signaling cascade;GO:0002115 store-operated calcium entry
CACNA1H	8912	CACNA1H	calcium voltage-gated channel subunit alpha1 H	GO:2000344 positive regulation of acrosome reaction;GO:0032342 aldosterone biosynthetic process;GO:0034651 cortisol biosynthetic process
GTPBP1	9567	GTPBP1	GTP binding protein 1	GO:0046039 GTP metabolic process;GO:0061014 positive regulation of mRNA catabolic process;GO:1901068 guanosine-containing compound metabolic process
NXPH2	11249	NXPH2	neurexophilin 2	GO:0007218 neuropeptide signaling pathway;GO:0007186 G protein-coupled receptor signaling pathway;GO:0007165 signal transduction
ENC1	8507	ENC1	ectodermal-neural cortex 1	GO:0010499 proteasomal ubiquitin-independent protein catabolic process;GO:0017148 negative regulation of translation;<br>GO:0034249 negative regulation of cellular amide metabolic process
NEURL3	93082	NEURL3	neuralized E3 ubiquitin protein ligase 3	GO:0016567 protein ubiquitination;GO:0032446 protein modification by small protein conjugation;<br>GO:0070647 protein modification by small protein conjugation or removal
HNRNPF	3185	HNRNPF	heterogeneous nuclear ribonucleoprotein F	GO:0035722 interleukin-12-mediated signaling pathway;GO:0071349 cellular response to interleukin-12;GO:0070671 response to interleukin-12
PITPNC1	26207	PITPNC1	phosphatidylinositol transfer protein cytoplasmic 1	GO:0015914 phospholipid transport;GO:0015748 organophosphate ester transport;GO:0006869 lipid transport
TRIM2	23321	TRIM2	tripartite motif containing 2	GO:0043161 proteasome-mediated ubiquitin-dependent protein catabolic process;GO:0043523 regulation of neuron apoptotic process;<br>GO:0000209 protein polyubiquitination
BAG2	9532	BAG2	BCL2 associated athanogene 2	GO:1904667 negative regulation of ubiquitin protein ligase activity;GO:0051444 negative regulation of ubiquitin-protein transferase activity;<br>GO:1904666 regulation of ubiquitin protein ligase activity
KCNIP4	80333	KCNIP4	potassium voltage-gated channel<br>interacting protein 4	GO:1901379 regulation of potassium ion transmembrane transport;GO:0061337 cardiac conduction;GO:0043266 regulation of potassium ion transport
RAG1AP1	55974	SLC50A1	solute carrier family 50 member 1	GO:0042946 glucoside transport;GO:1901656 glycoside transport;GO:0008645 hexose transmembrane transport
ZFHX4	79776	ZFHX4	zinc finger homeobox 4	GO:0000122 negative regulation of transcription by RNA polymerase II;GO:0045892 negative regulation of transcription, DNA-templated;<br>GO:1903507 negative regulation of nucleic acid-templated transcription
DIP2C	22982	DIP2C	disco interacting protein 2 homolog C	GO:0008150 biological_process
LGI4	163175	LGI4	leucine rich repeat LGI family member 4	GO:0022011 myelination in peripheral nervous system;GO:0014044 Schwann cell development;GO:0031641 regulation of myelination
EIF4G1	1981	EIF4G1	eukaryotic translation initiation factor 4 gamma 1	GO:1905618 positive regulation of miRNA mediated inhibition of translation;GO:1905612 positive regulation of mRNA cap binding;<br>GO:1905537 positive regulation of eukaryotic translation initiation factor 4F complex assembly
RICH2	9912	ARHGAP44	Rho GTPase activating protein 44	GO:0099152 regulation of neurotransmitter receptor transport, endosome to postsynaptic membrane;<br>GO:0098887 neurotransmitter receptor transport, endosome to postsynaptic membrane;GO:0099639 neurotransmitter receptor transport, endosome to plasma membrane
WIPI1	55062	WIPI1	WD repeat domain, phosphoinositide interacting 1	GO:0048203 vesicle targeting, trans-Golgi to endosome;GO:0034497 protein localization to phagophore assembly site;<br>GO:0048199 vesicle targeting, to, from or within Golgi
ZMYND15	84225	ZMYND15	zinc finger MYND-type containing 15	GO:0007286 spermatid development;GO:0048515 spermatid differentiation;GO:0007283 spermatogenesis
RNF220	55182	RNF220	ring finger protein 220	GO:0090263 positive regulation of canonical Wnt signaling pathway;GO:0051865 protein autoubiquitination;GO:0030177 positive regulation of Wnt signaling pathway
MATN2	4147	MATN2	matrilin 2	GO:0003429 growth plate cartilage chondrocyte morphogenesis;GO:0003414 chondrocyte morphogenesis involved in endochondral bone morphogenesis;<br>GO:0031104 dendrite regeneration
LPAR6	10161	LPAR6	lysophosphatidic acid receptor 6	GO:0051482 positive regulation of cytosolic calcium ion concentration involved in phospholipase C-activating G protein-coupled signaling pathway;<br>GO:0001835 blastocyst hatching;GO:0035025 positive regulation of Rho protein signal transduction
IBTK	25998	IBTK	inhibitor of Bruton tyrosine kinase	GO:0051209 release of sequestered calcium ion into cytosol;GO:0097553 calcium ion transmembrane import into cytosol;<br>GO:0061099 negative regulation of protein tyrosine kinase activity

GARS	2617	GARS1	glycyl-tRNA synthetase 1	GO:0070150 mitochondrial glycyl-tRNA aminoacylation;GO:0006426 glycyl-tRNA aminoacylation;GO:0015966 diadenosine tetraphosphate biosynthetic process
CDC42EP1	11135	CDC42EP1	CDC42 effector protein 1	GO:0031274 positive regulation of pseudopodium assembly;GO:0031272 regulation of pseudopodium assembly;GO:0031269 pseudopodium assembly
HMSD	284293	HMSD	histocompatibility minor serpin domain containing	GO:0010951 negative regulation of endopeptidase activity;GO:0010466 negative regulation of peptidase activity;GO:0045861 negative regulation of proteolysis
CSNK1D	1453	CSNK1D	casein kinase 1 delta	GO:1905426 positive regulation of Wnt-mediated midbrain dopaminergic neuron differentiation;GO:1905424 regulation of Wnt-mediated midbrain dopaminergic neuron differentiation; GO:1904958 positive regulation of midbrain dopaminergic neuron differentiation
SLC39A2	29986	SLC39A2	solute carrier family 39 member 2	GO:0071577 zinc ion transmembrane transport;GO:0006829 zinc ion transport;GO:0000041 transition metal ion transport
PIK3CD	5293	PIK3CD	phosphatidylinositol-4,5-bisphosphate 3-kinase catalytic subunit delta	GO:0033031 positive regulation of neutrophil apoptotic process;GO:0060374 mast cell differentiation;GO:0033029 regulation of neutrophil apoptotic process
SHC4	399694	SHC4	SHC adaptor protein 4	GO:0048863 stem cell differentiation;GO:0008284 positive regulation of cell proliferation;GO:0006915 apoptotic process
NALCN	259232	NALCN	sodium leak channel, non-selective	GO:0060075 regulation of resting membrane potential;GO:0035725 sodium ion transmembrane transport;GO:0071805 potassium ion transmembrane transport
CTDSPL	10217	CTDSPL	CTD small phosphatase like	GO:2000134 negative regulation of G1/S transition of mitotic cell cycle;GO:1902807 negative regulation of cell cycle G1/S phase transition; GO:2000045 regulation of G1/S transition of mitotic cell cycle
AHNAK	79026	AHNAK	AHNAK nucleoprotein	GO:1901385 regulation of voltage-gated calcium channel activity;GO:1901019 regulation of calcium ion transmembrane transporter activity; GO:1903169 regulation of calcium ion transmembrane transport
LGALS1	3956	LGALS1	galectin 1	GO:0002317 plasma cell differentiation;GO:0034120 positive regulation of erythrocyte aggregation;GO:0046598 positive regulation of viral entry into host cell
RDH5	5959	RDH5	retinol dehydrogenase 5	GO:0042572 retinol metabolic process;GO:0001523 retinoid metabolic process;GO:0016101 diterpenoid metabolic process
IRF8	3394	IRF8	interferon regulatory factor 8	GO:0032735 positive regulation of interleukin-12 production;GO:0042832 defense response to protozoan; GO:0060333 interferon-gamma-mediated signaling pathway
PDE4A	5141	PDE4A	phosphodiesterase 4A	GO:0006198 cAMP catabolic process;GO:0009214 cyclic nucleotide catabolic process;GO:0009154 purine ribonucleotide catabolic process
PRKCE	5581	PRKCE	protein kinase C epsilon	GO:0035669 TRAM-dependent toll-like receptor 4 signaling pathway;GO:2001031 positive regulation of cellular glucuronidation; GO:0035668 TRAM-dependent toll-like receptor signaling pathway
FOXP2	93986	FOXP2	forkhead box P2	GO:0021757 caudate nucleus development;GO:0021758 putamen development; GO:0060501 positive regulation of epithelial cell proliferation involved in lung morphogenesis
YWHAE	7531	YWHAE	tyrosine 3-monooxygenase/tryptophan 5-monooxygenase activation protein epsilon	GO:1900740 positive regulation of protein insertion into mitochondrial membrane involved in apoptotic signaling pathway;GO:1905913 negative regulation of calcium ion export across plasma membrane; GO:0003064 regulation of heart rate by hormone
NR5A2	2494	NR5A2	nuclear receptor subfamily 5 group A member 2	GO:0045070 positive regulation of viral genome replication;GO:0061113 pancreas morphogenesis;GO:1903902 positive regulation of viral life cycle
HOXA5	3202	HOXA5	homeobox A5	GO:0060480 lung goblet cell differentiation;GO:0060574 intestinal epithelial cell maturation;GO:0060481 lobar bronchus epithelium development
PRMT8	56341	PRMT8	protein arginine methyltransferase 8	GO:0019919 peptidyl-arginine methylation, to asymmetrical-dimethyl arginine;GO:0035247 peptidyl-arginine omega-N-methylation ;GO:0035246 peptidyl-arginine N-methylation
C11orf49	79096	C11orf49	chromosome 11 open reading frame 49	
RAB20	55647	RAB20	RAB20, member RAS oncogene family	GO:0090383 phagosome acidification;GO:0090385 phagosome-lysosome fusion;GO:0051452 intracellular pH reduction
HMGA2	8091	HMGA2	high mobility group AT-hook 2	GO:2000685 positive regulation of cellular response to X-ray;GO:0035978 histone H2A-S139 phosphorylation; GO:0045869 negative regulation of single stranded viral RNA replication via double stranded DNA intermediate
VARS2	57176	VARS2	valyl-tRNA synthetase 2, mitochondrial	GO:0006438 valyl-tRNA aminoacylation;GO:0106074 aminoacyl-tRNA metabolism involved in translational fidelity; GO:0006418 tRNA aminoacylation for protein translation
ZMIZ1	57178	ZMIZ1	zinc finger MIZ-type containing 1	GO:0007296 vitellogenesis;GO:0045582 positive regulation of T cell differentiation;GO:0048096 chromatin-mediated maintenance of transcription
ZNF704	619279	ZNF704	zinc finger protein 704	GO:0006357 regulation of transcription by RNA polymerase II;GO:0006366 transcription by RNA polymerase II; GO:0006355 regulation of transcription, DNA-templated
CTNND1	1500	CTNND1	catenin delta 1	GO:0035635 entry of bacterium into host cell;GO:0030260 entry into host cell;GO:0051806 entry into cell of other organism involved in symbiotic interaction
FARP1	10160	FARP1	FERM, ARH/RhoGEF and pleckstrin domain protein 1	GO:0010923 negative regulation of phosphatase activity;GO:0048813 dendrite morphogenesis;GO:0035305 negative regulation of dephosphorylation
PDE1A	5136	PDE1A	phosphodiesterase 1A	GO:0046069 cGMP catabolic process;GO:0009214 cyclic nucleotide catabolic process;GO:0009154 purine ribonucleotide catabolic process
ANKRD10	55608	ANKRD10	ankyrin repeat domain 10	
EPHB2	2048	EPHB2	EPH receptor B2	GO:1904782 negative regulation of NMDA glutamate receptor activity;GO:0048170 positive regulation of long-term neuronal synaptic plasticity; GO:0099557 trans-synaptic signaling by trans-synaptic complex, modulating synaptic transmission
ADRBK1	156	GRK2	G protein-coupled receptor kinase 2	GO:0003108 negative regulation of the force of heart contraction by chemical signal;GO:0003057 regulation of the force of heart contraction by chemical signal; GO:0045988 negative regulation of striated muscle contraction
FAM60A	58516	SINHCAF	SIN3-HDAC complex associated factor	GO:0030336 negative regulation of cell migration;GO:2000146 negative regulation of cell motility;GO:0051271 negative regulation of cellular component movement

GOLSYN	55638	SYBU	syntabulin	GO:1990048 anterograde neuronal dense core vesicle transport;GO:0099519 dense core granule cytoskeletal transport; GO:0035774 positive regulation of insulin secretion involved in cellular response to glucose stimulus
PPIL6	285755	PPIL6	peptidylprolyl isomerase like 6	GO:0000413 protein peptidyl-prolyl isomerization;GO:0042026 protein refolding;GO:0018208 peptidyl-proline modification
NRXN3	9369	NRXN3	neurexin 3	GO:0007158 neuron cell-cell adhesion;GO:0071625 vocalization behavior;GO:0007411 axon guidance
C6orf62	81688	C6orf62	chromosome 6 open reading frame 62	GO:0008150 biological_process
SNORA38	677820	SNORA38	small nucleolar RNA, H/ACA box 38	GO:0006396 RNA processing;GO:0016070 RNA metabolic process;GO:0090304 nucleic acid metabolic process
SULT2A1	6822	SULT2A1	sulfotransferase family 2A member 1	GO:0030573 bile acid catabolic process;GO:0050427 3'-phosphoadenosine 5'-phosphosulfate metabolic process;GO:0006068 ethanol catabolic process
PXT1	222659	PXT1	peroxisomal testis enriched protein 1	GO:0043065 positive regulation of apoptotic process;GO:0043068 positive regulation of programmed cell death;GO:0010942 positive regulation of cell death
MON1A	84315	MON1A	MON1 homolog A, secretory trafficking associated	GO:0006623 protein targeting to vacuole;GO:0072666 establishment of protein localization to vacuole;GO:0072665 protein localization to vacuole
FJX1	24147	FJX1	four-jointed box kinase 1	GO:0010842 retina layer formation;GO:0060042 retina morphogenesis in camera-type eye;GO:0003407 neural retina development
AGPAT9	79888	LPCAT1	lysophosphatidylcholine acyltransferase 1	GO:2001246 negative regulation of phosphatidylcholine biosynthetic process;GO:2001245 regulation of phosphatidylcholine biosynthetic process; GO:0036148 phosphatidylglycerol acyl-chain remodeling
FAM63A	55793	MINDY1	MINDY lysine 48 deubiquitinase 1	GO:0071108 protein K48-linked deubiquitination;GO:0016579 protein deubiquitination;GO:0070646 protein modification by small protein removal
STARD7	56910	STARD7	StAR related lipid transfer domain containing 7	GO:0006656 phosphatidylcholine biosynthetic process;GO:0046470 phosphatidylcholine metabolic process;GO:0015914 phospholipid transport
HINT2	84681	HINT2	histidine triad nucleotide binding protein 2	GO:2000757 negative regulation of peptidyl-lysine acetylation;GO:1901984 negative regulation of protein acetylation; GO:2000756 regulation of peptidyl-lysine acetylation
EMP1	2012	EMP1	epithelial membrane protein 1	GO:0032060 bleb assembly;GO:0008544 epidermis development;GO:0120031 plasma membrane bounded cell projection assembly
PDE1C	5137	PDE1C	phosphodiesterase 1C	GO:0007165 signal transduction;GO:0023052 signaling;GO:0007154 cell communication
FLJ32810		None	None	None
FGFBP1	9982	FGFBP1	fibroblast growth factor binding protein 1	GO:1903589 positive regulation of blood vessel endothelial cell proliferation involved in sprouting angiogenesis;GO:0090050 positive regulation of cell migration involved in sprouting angiogenesis;GO:1903587 regulation of blood vessel endothelial cell proliferation involved in sprouting angiogenesis
NUMA1	4926	NUMA1	nuclear mitotic apparatus protein 1	GO:1902365 positive regulation of protein localization to spindle pole body;GO:1902846 positive regulation of mitotic spindle elongation; GO:1902363 regulation of protein localization to spindle pole body
WDR47	22911	WDR47	WD repeat domain 47	GO:0007275 multicellular organism development;GO:0048856 anatomical structure development;GO:0032502 developmental process
TNKS1BP1	85456	TNKS1BP1	tankyrase 1 binding protein 1	GO:0006977 DNA damage response, signal transduction by p53 class mediator resulting in cell cycle arrest; GO:0072431 signal transduction involved in mitotic G1 DNA damage checkpoint;GO:0031571 mitotic G1 DNA damage checkpoint
RSPH4A	345895	RSPH4A	radial spoke head component 4A	GO:0060294 cilium movement involved in cell motility;GO:0060285 cilium-dependent cell motility;GO:0035082 axoneme assembly
ABCA1	19	ABCA1	ATP binding cassette subfamily A member 1	GO:0090108 positive regulation of high-density lipoprotein particle assembly;GO:0090107 regulation of high-density lipoprotein particle assembly; GO:0010875 positive regulation of cholesterol efflux
FLT1	2321	FLT1	fms related tyrosine kinase 1	GO:0036323 vascular endothelial growth factor receptor-1 signaling pathway;GO:1905563 negative regulation of vascular endothelial cell proliferation; GO:0030949 positive regulation of vascular endothelial growth factor receptor signaling pathway
EMP2	2013	EMP2	epithelial membrane protein 2	GO:0003093 regulation of glomerular filtration;GO:0070836 caveola assembly;GO:2001212 regulation of vasculogenesis
VPS25	84313	VPS25	vacuolar protein sorting 25 homolog	GO:0007175 negative regulation of epidermal growth factor-activated receptor activity;GO:0043328 protein transport to vacuole involved in ubiquitin-dependent protein catabolic process via the multivesicular body sorting pathway;GO:0007176 regulation of epidermal growth factor-activated receptor activity
SEPW1	6415	SELENOW	selenoprotein W	GO:0010269 response to selenium ion;GO:0098869 cellular oxidant detoxification;GO:1990748 cellular detoxification
CYP4F12	66002	CYP4F12	cytochrome P450 family 4 subfamily F member 12	GO:0003095 pressure natriuresis;GO:0036101 leukotriene B4 catabolic process;GO:0036100 leukotriene catabolic process
C6orf47	57827	C6orf47	chromosome 6 open reading frame 47	
BTBD18	643376	BTBD18	BTB domain containing 18	GO:1990511 piRNA biosynthetic process;GO:0007141 male meiosis I;GO:0032968 positive regulation of transcription elongation from RNA polymerase II promoter
FOXJ3	22887	FOXJ3	forkhead box J3	GO:0045944 positive regulation of transcription by RNA polymerase II;GO:0045893 positive regulation of transcription, DNA-templated GO:1903508 positive regulation of nucleic acid-templated transcription
CAMK1D	57118	CAMK1D	calcium/calmodulin dependent protein kinase ID	GO:0090023 positive regulation of neutrophil chemotaxis;GO:0071624 positive regulation of granulocyte chemotaxis GO:0090022 regulation of neutrophil chemotaxis
C4orf51	646603	C4orf51	chromosome 4 open reading frame 51	
RANBP3L	202151	RANBP3L	RAN binding protein 3 like	GO:1901706 mesenchymal cell differentiation involved in bone development;GO:0045663 positive regulation of myoblast differentiation; GO:0045668 negative regulation of osteoblast differentiation

COL21A1	81578	COL21A1	collagen type XXI alpha 1 chain	GO:0003429 growth plate cartilage chondrocyte morphogenesis;GO:0003414 chondrocyte morphogenesis involved in endochondral bone morphogenesis; GO:0003422 growth plate cartilage morphogenesis

Supplemental Figure S1. Venn diagram showing the proportion of genome-wide coverage of 135 differentially methylated CpG sites. Promoters, gene bodies, intergenic regions, and gene 3' ends are shown (left panel). The differentially methylated CpG sites were annotated according to CpG islands, CpG shores (2-kb regions flanking CpG islands) and CpG shelves (2-kb regions flanking CpG shores) (right panel).

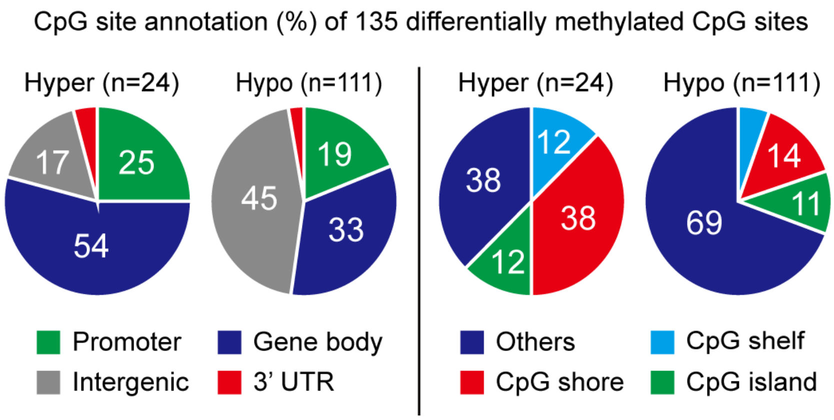

Supplemental Figure S2. Methylation analysis of the *FHIT* promoter regions in patient with CD samples. Gel-based MSP results of *FHIT* gene (related to Figure 3b). We tested 207 patient with CD samples but here we show the representative results (n=36 CD samples) to related Figure 3b in the text. The PCR products distinguish between unmethylated (U) and methylated (M). Gray circle indicated we count on unmethylated samples but rest of samples showed methylation signal. DKO cells were used as an unmethylated control. IVD: in-vitro methylated control, H_2O: water control containing no DNA.

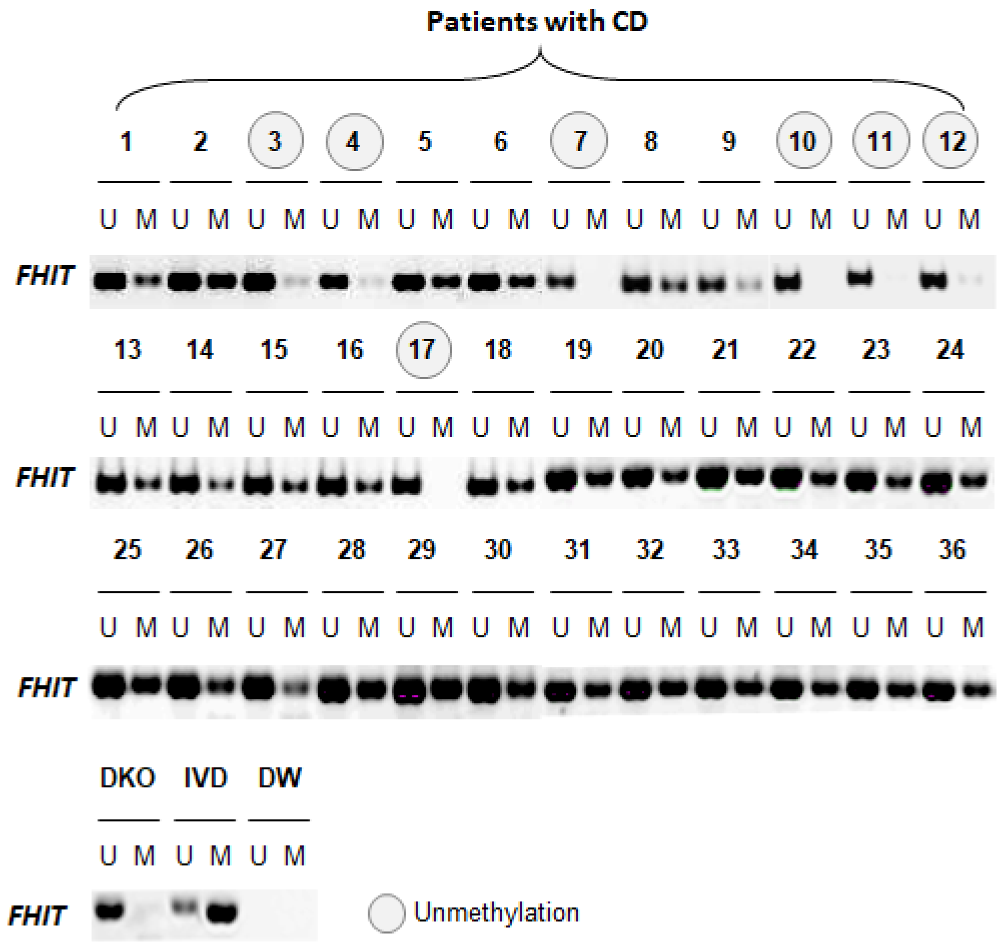

Supplemental Figure S3. Biological functional implication of hypermethylated genes in CD. GO analysis was conducted using Metascape. Each heatmap of the implicated biological pathways indicates those pathways significantly associated with the genes in the network.

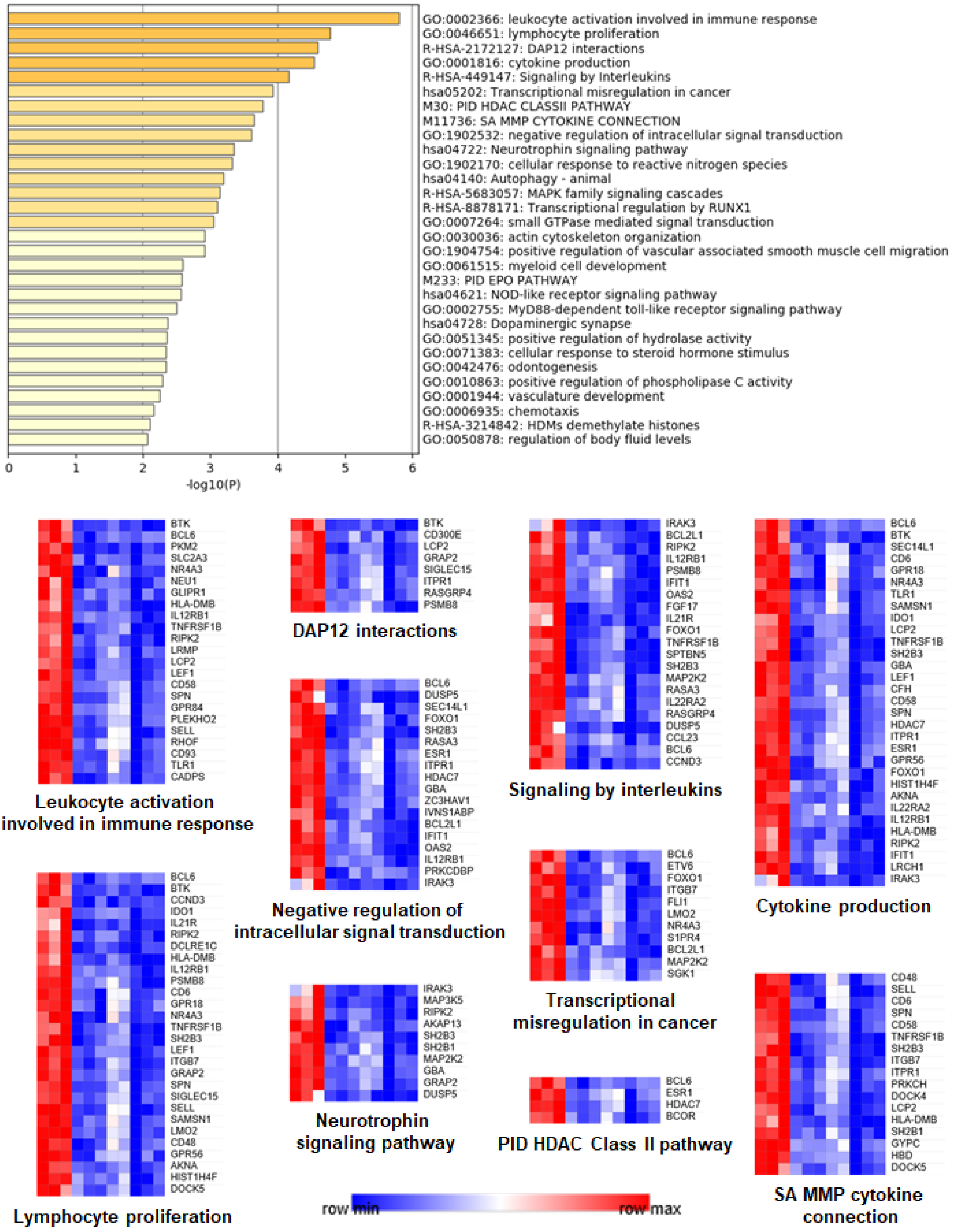

Supplemental Figure S4. Implication of the clinical relevance of *FHIT* gene methylation in patients with CD. Kaplan-Meier survival curves for disease duration [over 5 years] from diagnosis to sampling based on *FHIT* methylation for 88 patients with CD. A probability of < 0.05 (log-rank test *p < 0.05) was considered to represent a statistically significant difference.

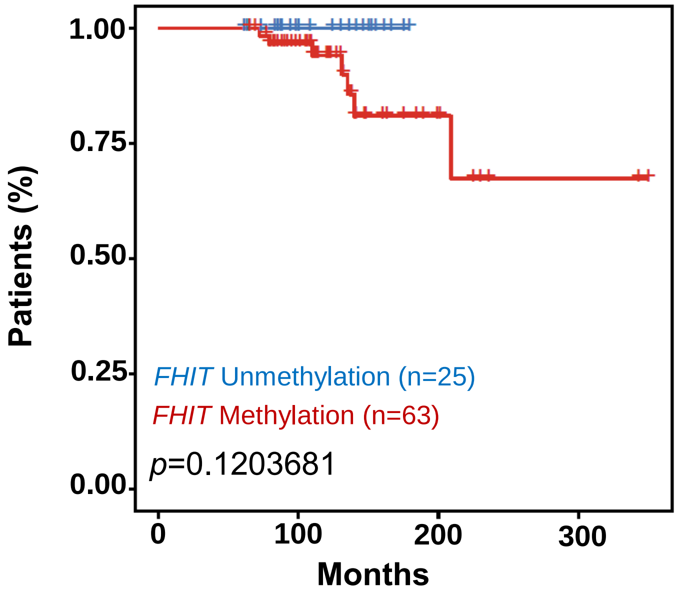

Supplement: Supplementary file 1 [file jcm-09-01338-s001.pdf]
